# Revisiting Wireless Cyberattacks on Vehicles

**DOI:** 10.3390/s25082605

**Published:** 2025-04-20

**Authors:** Roberto Gesteira-Miñarro, Gregorio López, Rafael Palacios

**Affiliations:** 1 Institute for Research in Technology, Comillas Pontifical University, 28015 Madrid, Spain; gllopez@comillas.edu (G.L.); palacios@mit.edu (R.P.); 2 Cybersecurity at MIT Sloan, Massachusetts Institute of Technology, 77 Massachusetts Avenue, Cambridge, MA 02139, USA

**Keywords:** cyberattacks, radio frequency, vehicles, wireless

## Abstract

The automotive industry has been a prime target for cybercriminals for decades, with attacks becoming more sophisticated as vehicles integrate advanced digital technologies. In response, new standards and regulations have been introduced, requiring manufacturers to implement robust cybersecurity measures to obtain necessary certifications. Modern vehicles have an extensive attack surface due to the increasing number of interconnected electronic components and wireless communication features. While new technologies improve connectivity, automation, and comfort, they also introduce new vulnerabilities that can be exploited by attackers. This paper presents a comprehensive analysis of the attack surface of modern vehicles, focusing on the security risks associated with wireless communication technologies. Each technology is examined in detail, highlighting existing research, known vulnerabilities, and potential countermeasures. Furthermore, this study identifies key research gaps in the field, providing insights into critical areas that require further investigation. This work aims to guide future research efforts in order to enhance vehicle cybersecurity in the evolving landscape of smart, autonomous, and connected vehicles.

## 1. Introduction

Modern vehicles are designed with many features to provide human safety and comfort. However, adding more functionalities can lead to security issues, since there are more assets to protect. As a result, new regulations have come into force in the automotive industry, and manufacturers must take cybersecurity into account to pass certain certifications (UN Regulation No. 155, 156) [1,2] before selling their products [3]. With the rise in autonomous vehicles, connected vehicles, shared vehicles, and vehicle-to-everything communications (V2X), an increase in research projects in this field is expected.

The automotive industry has been a target for cybercriminals for decades. There are many resources related to automotive cybersecurity (also known as “car hacking” in the hacker community). Nevertheless, most of the knowledge is kept in cybersecurity conferences rather than in academic papers. It is common to see talks about car hacking at cybersecurity conferences such as Black Hat (San Francisco, CA, USA) or DEF CON (Las Vegas, NV, USA). In fact, every year, there is a Car Hacking Village [4] (associated with DEF CON) that joins several researchers on this topic. In addition, there is the Automotive Security Research Group (ASRG) [5] (Stuttgart, Germany); or VicOne [6] (Tokio, Japan), which is a company that focuses on automotive cybersecurity and sponsors the Pwn2Own Automotive competition [7], where hackers from all over the world try to compromise some of the proposed targets for fame and bounties.

In this topic, private-industry researchers are more advanced than academic researchers. This is probably because of interest or budget reasons. In fact, many of the attacks and techniques covered in this paper are already implemented in hardware devices that can be purchased in underground forums in the Dark Web, probably illegal.

The peak of “car hacking” came when Miller and Valasek showed how to compromise a 2014 Jeep Cherokee [8] using different attack vectors in Black Hat USA 2015 [9]. The authors also wrote a white paper [10], which contains almost the same information, but in a more formal register. Although there are papers on automotive cybersecurity before, this milestone triggered a lot of research and cybersecurity awareness regarding vehicles. The same year, Samy Kamkar presented RollJam at DEF CON 23 [11], an attack to bypass rolling-code implementations on Remote Keyless Entry (RKE) systems.

In order to improve and test the security of existing and future vehicles, car manufacturers need to rely on research projects, to have an external perspective. This research is not only valuable for vehicle manufacturers, but also for insurance companies, since they need to estimate the insurance rates depending on different factors, such as, for instance, the security level of a given vehicle. In fact, there are insurance companies that nowadays are not willing to handle some car models due to their high rate of car theft [12]. Although the entry systems have several mitigation features, news about car theft is very frequent. Cybercriminals always find a way to bypass these protection mechanisms and exploit vulnerabilities. This is the reason why countries such as Canada have recently prohibited the use of hacking devices like Flipper Zero [13], which are known to be used for car hacking.

The attack surface of a vehicle is very wide, and keeps growing with the advances in technology. A car is composed of a large amount of Electronic Control Units (ECUs). These components are sensors and actuators that connect the car with the external environment. Each ECU has a specific functionality, and all of them are connected with each other via serial buses. The communication between the different ECUs is handled by the Controller Area Network (CAN) protocol [14]. The CAN bus uses an old protocol that was not designed with cybersecurity in mind. For instance, there is no encryption, no authentication, and the network has a bus topology. As a result, the CAN bus is a juicy target for cybercriminals to compromise a vehicle, because they can potentially eavesdrop CAN messages from the ECUs and send arbitrary CAN messages or commands to any ECU. These issues are already addressed in [15], as well as several ideas for Intrusion Detection Systems to mitigate attacks in [16] or [17].

Although the CAN bus is accessible through the On-Board Diagnostics port (OBD-II) for diagnosis purposes in a car workshop [15], it is also connected to the infotainment system in modern vehicles, which poses a higher risk and attack surface for cybercriminals to compromise a vehicle. CAN bus vulnerabilities are not likely to be corrected because communication between ECUs must be extremely fast, as human safety might be affected. Therefore, the CAN bus must be a fast network where latency is negligible. Because of that, there is no encryption or message authentication, to minimize processing times on the ECU side (as well as power consumption, among other characteristics). Therefore, it is the most critical target for attackers in order to compromise a vehicle. In this context, thieves have found a technique known as CAN injection [18] in order to obtain access to the CAN bus. They take a twisted pair cable inside the headlights and use it to connect directly to the CAN bus, so that they can read and inject arbitrary CAN messages [19].

With the advances in technologies like artificial intelligence, image processing or cellular communications, new concepts have appeared: autonomous vehicles, vehicle-to-vehicle (V2V) communications, vehicle-to-infrastructure (V2I) communications, and, in general, vehicle-to-everything (V2X) communications. These new advances in the automotive sector are being designed nowadays with cybersecurity and human safety in mind. It is important to keep these communications as fast as possible, minimizing latency, while making them secure and private. There are already papers that cover these topics in depth, such as [3], [20], or [21]. More specific security issues in V2X communications can be found in [22].

The consequences of performing a cyberattack on a vehicle can result in denial of service (DoS), car theft, personal-asset theft, information disclosure, cyberphysical damage, or even remote control, among others.

The purpose of this paper is to revisit existing attacks against vehicles that require the attacker to use wireless technologies near the car. We provide useful references and a list of research gaps in order to raise awareness and show the importance of research on these topics to improve the security of vehicles, because it will be relevant for future designs.

First, some useful tools are shown as a place to start with any research related to wireless technologies. Then, a literature review and the state of the art is presented and analyzed, including keyless entry systems and immobilizers. After that, other wireless technologies are documented. Finally, some research gaps and conclusions are drawn.

## 2. Radio-Frequency Tools

This section contains some tools that can be used to audit radio-frequency devices and perform cyberattacks. The list shows the available commercial hardware tools and software tools; all of them are open source.

### 2.1. Hardware

- HackRF One [23] (versions r1 through r10), from Great Scott Gadgets, is a half-duplex transceiver that operates at frequencies from 1 MHz to 6 GHz and can reach a rate of 20 million samples per second. With HackRF One and the appropriate software, the real-time frequency spectrum can be visualized. Moreover, it allows us to capture and replay signals.
- There is another device from Great Scott Gadgets, known as YARD Stick One [24], that accepts different types of digital modulations (ASK/OOK, GFSK, 2FSK, 4FSK, MSK) and rates of up to 500 kbps. This gadget can be used to capture and decode signals, and to generate synthetic signals from binary information.
- Proxmark 3 RDV4 [25] is a tool designed mainly for RFID analysis and research. It allows for testing, sniffing, replaying, and cloning devices such as RFID tags or Mifare Classic cards. Proxmark can be used to analyze immobilizers, which usually work as an RFID device. It can also be used to assess vehicles that have a PKES system to lock/unlock the car and even to start the engine, such as Tesla.

### 2.2. Software

- To interact with a HackRF One gadget, GNU Radio Companion [26] is a good option, which is a project that provides a graphical programming environment based on signal processing blocks for interacting with software-defined radio (SDR) devices.
- GQRX [27] is a program based on GNU Radio Companion that displays the frequency spectrum in a waterfall model and is able to apply signal processing to received radio signals. It can be used to identify the working frequency of a given key fob with HackRF One.
- inspectrum [28] is software that displays the power of a signal in time and frequency. It is used to analyze signal capture files and extract their characteristics and even encoded symbols as bits.
- Universal Radio Hacker [29] is a project that encompasses the previous functionalities: it can be used to send and receive radio signals, but also to analyze their encoded information.
- rfcat [30] (version 2.0.1) is a Python 3 library dedicated to the use of YARD Stick One.

## 3. State of the Art

This section presents works related to wireless cyberattacks on vehicles, including keyless entry systems (RKE and PKES), immobilizers, and other wireless technologies (TPMS, Bluetooth, and GPS).

### 3.1. Remote Keyless Entry

The keyless entry system of a car allows users to lock and unlock their vehicles, among other actions such as opening the trunk or starting up the engine remotely. The RKE system is simple from the user’s viewpoint, since they only need to press a button on their key fob, and the action is performed automatically. From the technical point of view, a digital signal is sent by the key fob and the car analyzes that signal and executes the requested action.

RKE systems are vulnerable to replay attacks and jamming by construction, due to how they work. Some tools to perform these attacks are presented in [31], but these are script-kiddie techniques, because the only requirement is to have the proper tools. In [32], a reverse-engineering approach is shown to analyze garage door openers. This perspective is more insightful, because all the protocol details are discovered, so that the attacks can be understood in a deeper way. A similar resource to learn about reverse engineering in radio-frequency protocols can be found in [33,34].

RKE signals are usually modulated with digital modulations. On the one hand, there are modulations such as Amplitude Shift Keying, particularly On-Off Keying (ASK/OOK). On the other hand, modulations such as Frequency Shift Keying exist, especially using two carrier frequencies (2FSK). In most cases, the signals start with a synchronization sequence and also use Manchester coding as a channel encoding, to prevent synchronization errors on the receiver. Furthermore, some key fobs will send the same information several times to ensure that the receiver receives the signal.

Most of the protocols used for keyless entry protocols are proprietary and closed source. However, this security by obscurity is usually beaten with information leaks in underground forums or reverse engineering. This is the case of KeeLoq, which is a block cipher owned by Microchip [35]. After that, a lot of research papers emerged and it was proven to be a weak cipher, with various cryptographic and side-channel attacks. Other keyless entry ciphers are Hitag2 [36] or AUT64 [37].

#### 3.1.1. Replay Attacks

A vehicle will unlock whenever a valid signal is received, thus showing that RKE systems have an intrinsic vulnerability, because it the device that sends the signal does not matter. As a result, if an attacker manages to capture a valid signal, they might be able to unlock the car by sending the captured signal (replay attack) [32].

Rolling codes were introduced to mitigate replay attacks in RKE systems. These codes can only be used once. The idea is that the key fob and the vehicle have a pseudorandom number generator (PRNG) initialized with the same seed, so that the key fob sends the next step of the algorithm and the vehicle can match the code within a list of valid codes. Consequently, once a code is used, it is marked as invalid to prevent replay attacks.

Other rolling-code implementations make use of symmetric ciphers like Hitag2 or KeeLoq. With this approach, the key fob holds a counter that is increased on every button press. This counter is encrypted along with other fields such as key identifiers and sent along with the encoded command. The vehicle is able to decrypt the rolling code and determine if the counter is valid or not and advance it accordingly [38].

Regardless of the type of rolling-code implementation, it must be indistinguishable and not predictable for an adversary. In other words, given a rolling code, it must be impossible to tell if it comes from a PRNG or a cipher, even with multiple samples.

**Figure 1:**
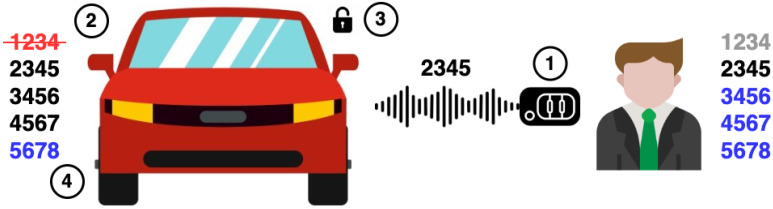
Operation of rolling codes.

Figure 1 illustrates the way rolling codes work, using a PRNG-based approach and dummy numbers that mimic rolling codes. The list of numbers at the right represents the PRNG status of the key fob, saying that code 1234 is used, 2345 is the next PRNG output, and the rest are future outputs. To the left, we have the list of rolling codes: 1234 is discarded because it is already used; 2345, 3456, 4567 are valid rolling codes; and 5678 will be added later. Following the steps in the figure, (1) the user presses a button on the key fob to open the car. The corresponding signal holds rolling code 2345. Then, (2) the vehicle checks if the rolling code is within the list of valid codes. If not, the vehicle must ignore the request. If the code is valid, the code is marked as invalid (just like 1234) and (3) the action is performed. After that, (4) the vehicle adds a new valid code to the list, namely 5678. A similar procedure can be implemented for cipher-based rolling codes. Instead of having a list of valid codes, the vehicle needs to decrypt the rolling code and tell if the encoded number is greater than its current counter. If so, the action is performed; if not, it means that the signal held a past counter value, so it must be discarded.

In PRNG-based rolling codes, the list of valid rolling codes must be sufficiently large. The aim is to preserve synchronization with the key fob, because the user may press the button by accident and waste some rolling codes that the vehicle has not received. Otherwise, the next PRNG output of the key fob would not be in the list and the car will not perform the requested action.

Nevertheless, there are still ways to perform replay attacks. The limitation is that the vehicle must not receive the code. Therefore, if an attacker captures a signal with a valid code from the key fob and the car is not near, they will be able to replay the signal and the car will behave normally because the code is treated as valid.

In some cases, a brute-force approach might be useful. There was a talk at DEF CON 32 [39] about a specific type of programmable rolling code, known as learning codes, that are only 20 bits long. The researcher showed how he was able to reverse-engineer the RKE protocol to find its structure and perform a 20-bit brute-force attack to unlock these cars.

#### 3.1.2. RollJam Attack

In relation to rolling codes, Samy Kamkar presented a new technique called RollJam [11] in DEF CON 23 to attack rolling-code implementations in RKE systems. The aim of the attack is to obtain a set of valid codes that can be used to unlock the car later.

**Figure 2:**
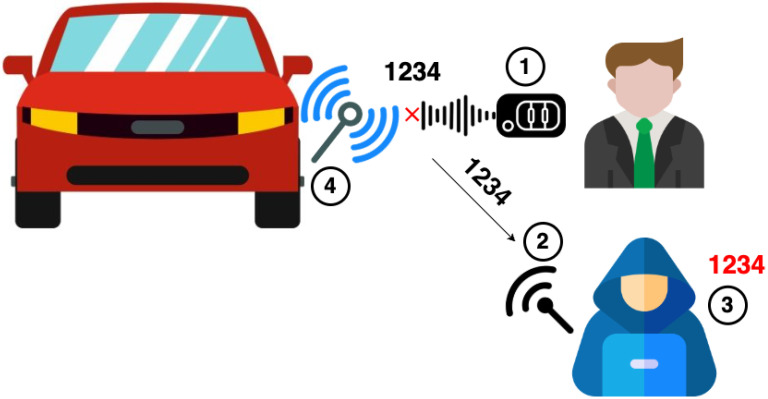
A RollJam attack scenario on an RKE system.

The attack involves using a jamming antenna (jammer) near the vehicle (i.e., under the vehicle to remain unnoticed) in order to generate interference in the working frequency band. As a result, the legitimate key fob will not work as expected because the car will not receive any signal. As shown in Figure 2, (1) the user will press the buttons several times, but the car will not perform any action due to interferences. Meanwhile, (2) the attacker can capture these failed rolling codes. The attacker must be next to the key fob to capture those signals, but far enough from the car to be outside the interference range. These codes (3) are valid because the car has not received any of them, so they are still in the list of valid codes (or hold a valid encrypted counter). Eventually, (4) the attacker should deactivate the jammer and use one of the first captured signals to unlock the car and make the victim think that the key fob is working again [11]. Otherwise, the user might open the car with the physical key, which ensures that all the captured codes can not be used; this method improves upon Samy Kamkar’s previous approach [31]. These valid codes grant access to the interior of the vehicle and potentially enable someone to steal personal belongings and information from the car owners and users.

Considering jamming techniques, in [40], it is discovered that RKE key fobs employ an envelope detector, which is much more vulnerable to pulsed electromagnetic interference than to continuous interference. The researchers show that the use of a synchronous detector mitigates the issue.

There are other rolling-code implementations that attempt to protect against replay and RollJam attacks, for instance, in [41]. On the other hand, ref. [42] proposes a protocol based on RSA signatures using a hash function to take the current date and the current time into account, as well as a random number generated with a PRNG using an increasing counter as a seed on both the key fob and the vehicle.

#### 3.1.3. RollBack Attack

Another vulnerability was found in rolling-code implementations, which gave birth to an attack known as RollBack [43], presented at Black Hat USA 2022. The researchers found that by sending two or more already used and consecutive signals, the vehicle will resynchronize the key fob and perform the encoded action.

This attack is much more dangerous, powerful, and easy to perform. The attacker just needs to capture two or more signals that the vehicle has received (the type of action through which they are obtained does not matter). The researchers showed a proof-of-concept video that proves how they were able to reuse this set of consecutive signals multiple times to unlock the vehicle. Nevertheless, not every vehicle brand is vulnerable to this attack.

### 3.2. Passive Keyless Entry and Start

**Figure 3:**
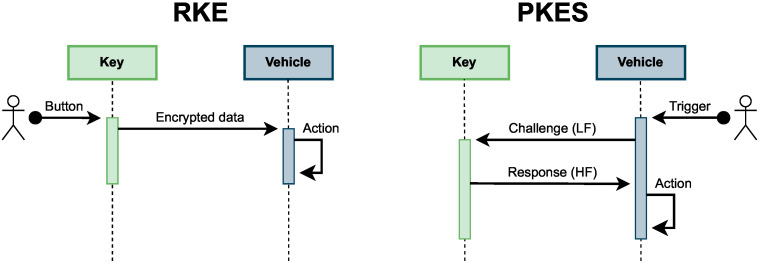
RKE and PKES message flows.

In Passive Keyless Entry and Start (PKES) systems, the user is not required to press a button on the key fob. They just need to approach the vehicle and the doors will unlock after bidirectional communication between the key fob and the vehicle [44]. Figure 3 illustrates the differences between RKE and PKES message flows.

The “Trigger” signal in PKES systems can be different depending on the manufacturer. There are vehicles that require the user to touch the door handle, whereas other vehicles send a beacon signal periodically and wait until a key fob responds with an ACK. In both cases, once the “Trigger” action is completed, the vehicle sends a cryptographic challenge which only the legitimate key fob configured in the car is supposed to solve. If the response from the key fob is correct, then the vehicle will perform the required action.

In PKES systems, the process of starting the engine is similar, there is no need to enter the key to switch on the engine. The car verifies that the key fob is inside the vehicle and the engine starts after the user has pressed the “Start” button (which is the “Trigger” signal in Figure 3).

#### Relay Attacks

**Figure 4:**
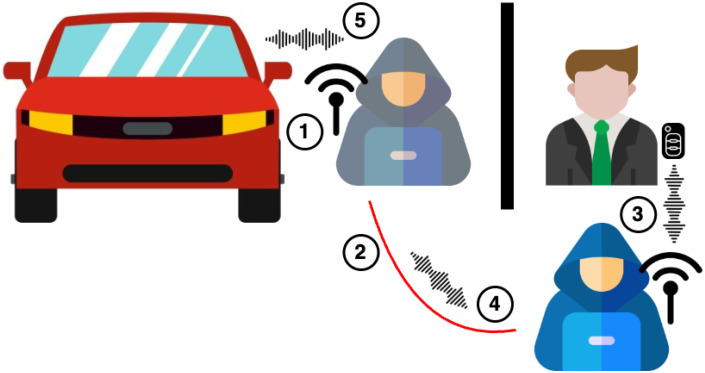
Relay attack scenario on a PKES system.

PKES systems are vulnerable to relay attacks due to the way the protocol works. The attack scenario requires two malicious attackers, one near the key fob and one next to the car. As Figure 4 shows, (1) the attacker who is next to the car sends the “Trigger” signal and (2) relays the “Challenge” signal to the attacker that is next to the key. Then, (3) the “Challenge” signal is amplified and the key fob responds (as if the signal was produced by the car). Finally, (4) the “Response” signal is relayed to the attacker next to the vehicle and amplified, so that (5) the vehicle unlocks the doors, since the response message is correct. Furthermore, the attackers can potentially start the engine, because the process is mostly the same. Reference [45] illustrates how the attack is performed.

In fact, relay attacks on PKES systems are dangerous because the attackers can potentially steal the car and leave. The devices required for the attack are not expensive, and the communication between the attackers can be either wired or wireless [46].

Regarding mitigations to relay attacks on PKES systems, in [47], the researchers propose the use of Bluetooth Low Energy (BLE) and several parameters like RSSI, RTT, GPS, or Wi-Fi access-point lists to precisely determine whether the key fob is close to the vehicle or not. Basically, the key fob is the prover and the vehicle is the verifier. The prover must send the aforementioned parameters, so that the verifier can measure distances and geographical position. Although the protocol looks secure enough, we believe that if the attacker can send the expected information through BLE, they could fool the verifier and still be able to perform a successful relay attack.

On the other hand, ref. [44] proposes radio-frequency fingerprinting. They basically train a classification model with legitimate signals from different key fobs, so that malicious signals sent from a radio dongle or an SDR device can be detected. This mitigation is more robust, because car manufacturers can train their own models in order to define a baseline that contains all the radio-frequency features of their devices, making it difficult for an attacker to bypass this anomaly-based countermeasure.

In [48], they use a timestamp so that, if the car receives an old signal, it discards the code and will not perform any action. This approach is also correct as long as the time measurement is precise enough to prevent de-synchronization or other side effects.

### 3.3. Immobilizer

An immobilizer system is an antitheft device that protects the ability to start the engine. It requires cryptographic authentication from a Radio-Frequency IDentification (RFID) transponder embedded within the key fob, so that it prevents an attacker from hot-wiring a car. In Europe, the immobilizer system has been mandatory since 1995 [49].

As in RKE and PKES systems, the majority of immobilizer cryptography protocols are proprietary. In [49], the researchers extracted the firmware from the immobilizer and reverse-engineered the cryptographic algorithm, which is called Digital Signature Transponder 80 (DST80), from Texas Instruments. They presented some examples where they could recover the cryptographic key and disable the transponder. Moreover, they found a way to recover the full 80-bit key using side-channel techniques.

Megamos Crypto is another protocol for immobilizer systems, analyzed in [50]. Again, the researchers extracted and reverse-engineered an ECU firmware and found weaknesses in the cryptographic algorithm that could lead to the recovery of the 96-bit key.

Hitag2 is also used as a cryptographic algorithm for immobilizer systems. In [51], the researchers performed a detailed analysis of the Hitag2 cryptographic algorithm (designed by NXP), which is used by some manufacturers for rolling-code RKE systems as well as RFID immobilizer protocols. They successfully found a way to break the Hitag2 cipher with a few samples. Furthermore, in [36], the researchers found a way to compromise the cipher in around 6 min with commercial hardware. Further analyses of the Hitag2 cryptosystem for RFID immobilizers and RKE systems can be found in [50,52]. An OpenCL implementation to break Hitag2 is described in [53]. Compared to FPGA implementations, it is not efficient, but it is less expensive. In the worst case, they successfully break the cipher after 11 h. An optimized guess-and-determine attack is proposed in [54] to break Hitag2. They claim that their implementation is able to recover the cryptographic key with 100% success rate and only two RKE signal samples.

In [55], a reverse-engineering process is shown to compromise the PKES system and the immobilizer in luxury vehicles. They show the use of an inadequate proprietary cipher with 40-bit keys and the lack of mutual authentication in the challenge–response protocol.

### 3.4. Tire-Pressure Monitoring System

The Tire-Pressure Monitoring System (TPMS) has been investigated in [56]. These systems continuously measure air pressure inside all tires of a vehicle, and alert drivers if any tire is significantly underinflated. Although it is a safe-critical application, it can be misused in two ways:

- It can be used to track a certain vehicle, because it is an automatic protocol and difficult to deactivate.
- TPMS signals can be easily jammed or spoofed, because they use radio-frequency communications. It could lead to false dashboard warnings.

The TPMS protocol is very similar to RKE/PKES systems, because it uses the same frequency band (433 MHz in Europe and 315 MHz in the United States), modulation (2FSK and ASK/OOK), and encoding (Manchester). Furthermore, reverse engineering is needed to understand the protocol [56]. After that, it is possible to eavesdrop TPMS messages and even send arbitrary messages.

### 3.5. Bluetooth

Bluetooth is another wireless communication protocol that is being used lately for several car functionalities. It uses the frequency band of 2.4 GHz and a distance range of up to 10 m (although it can be extended to 100 m). There have been several cryptographic designs on top of Bluetooth. However, due to inefficiency, most manufacturers avoid using these protocols because of that [21].

One of the first Bluetooth cyberattacks was introduced in 2011 by Checkoway et al. [57]. The attacker manipulated the vehicle radio system using a weakness in the Bluetooth stack due to the use of insecure functions such as strcpy in the C programming language [58]. Once the radio system was compromised, they gained access to the CAN network and sent messages to an ECU to disable the brakes.

In relation to the CAN bus, there are Bluetooth dongles that connect to the OBD-II port and communicate with a mobile application to show vehicle diagnostics and statistics to the end-user. It is interesting to analyze these types of functionalities to test if the Bluetooth connection is secure. In [59], it is shown how a malicious mobile app can interact with the OBD-II connector and perform actions on the CAN bus. The researchers show a reverse-engineering methodology to analyze CAN messages in order to find the protocol structure; afterwards, the mobile application can receive instructions from a back-end server and perform malicious actions on the car via CAN messages (engine, brakes, wheel, etc.). They show a proof-of-concept video where the researchers control a vehicle remotely from their mobile phone (which is connected via Bluetooth with the OBD-II connector).

Bluetooth is also used for the keyless entry system of vehicles like Tesla. In [60], the researchers show how they were able to exploit new attack vectors due to the use of Bluetooth. Particularly, the Tesla Model X uses Bluetooth Low Energy. The key fob exposed more attack vectors than traditional key fobs because of Over-The-Air firmware updates and pairing functionality of new keys.

The uses of Bluetooth on car subsystems can have a huge impact. For instance, an adversary could gain access to the car if Bluetooth is used for keyless entry systems, and probably start the engine because modern vehicles implement PKES. On the other hand, an attacker could gain access to a CAN bus through an OBD-II connector.

In addition, car-sharing platforms rely on mobile applications that let users unlock their cars with a mobile application that connects via Bluetooth. If this communication is not secure enough, an adversary could find the flaws and exploit them to obtain unauthorized access to car-sharing vehicles. In [61], the authors present a novel physical keyless car-sharing system that allows car owners to generate digital keys for accessing their cars, and to share these keys with other users. It also provides a comprehensive analysis on the threats of car-sharing systems.

### 3.6. Global Positioning System

The Global Positioning System (GPS) is finding relevance in the context of V2V and V2X communications, since the entities involved in the dialogue must know the exact position of each other [62].

As well as other wireless radio-frequency technologies, GPS is vulnerable to jamming and spoofing, since GPS signals do not contain any information that can authenticate the source of the signals [58]. Several techniques for jamming GPS systems are presented in [63], using commercial SDR devices such as ADALM-Pluto, BladeRF, USRP, or HackRF One and open-source software such as GNU Radio Companion. A curated list of attack scenarios to GPS as well as defenses and mitigations are shown in [58]. They describe the attack scenario, the criteria for defense strategies, and existing defense strategies for both spoofing and jamming attacks on GPS.

Regarding detection mechanisms for GPS spoofing attacks, ref. [21] shows several strategies, such as a bias-estimation range check, velocity consistency check, statistical test, least absolute shrinkage and selection operator, and global navigation satellite system augmentation.

## 4. Research Gaps

Although there are already a considerable amount of research papers written on automotive cybersecurity and wireless attacks, there are still topics that can be further investigated and topics that are emerging nowadays because of the use of modern technologies.

- Risk assessment: The risks and impact of automobiles need to be measured. On the one hand, new regulations force manufacturers to pass cybersecurity tests [3]. On the other hand, insurance companies must take this variable into account when defining the policy. Although risk assessment in the automotive sector is very complex, some approximations using well-known frameworks can be found in [64].
- Supply chain: The automotive industry employs a complex supply chain to source the components that are used to build new vehicles, provide services and perform repairs. This supply chain poses a huge risk to the industry, since each connected endpoint is a vulnerability waiting to happen [65]. It is a fact that the security of individual components does not ensure the security of the whole system.
- Reverse engineering: Vehicle manufacturers will never publicly disclose any source code or detailed specification for the products they build. For this reason, researchers must utilize their reverse-engineering skills to extract ECUs firmware and analyze compiled artifacts from closed-source microprocessors, for example. Others might want to analyze radio signals or discover logic bugs in state-machine systems.
- Cryptography: On the one hand, there are protocols like RKE, PKES, and TPMS that still lack robustness. Modern cryptographic schemes must be considered, such as lightweight cryptography [66]. On the other hand, for devices that have no strict computational power limitation, post-quantum algorithms are preferred to prevent traditional algorithms to be broken with quantum computers.
- Mitigations: While there is research about vulnerabilities and methods to compromise vehicular technologies, there must also be research about how to defend from these techniques and protect the affected systems.
- Software security: Modern cars are equipped with many features that improve the user experience and comfort. However, these new systems integrated in the car, such as operating systems or web browsers, must be analyzed. Manufacturers should follow best practices and continuous integration with quality assurance to prevent bugs and errors in their codebase.
- V2X communications: With the advances on technology, image processing, robotics and artificial intelligence, there habe been a lot of studies on autonomous vehicles. These vehicles must communicate with each other (V2V) and with other entities (V2X). Standards are still being designed [67]. As a result, there is a need to protect these communications from the design phase, because they can be critical for human safety. Modern post-quantum cryptographic protocols are taken into consideration for this application, as shown in [68,69,70].
- Digital twins: Digital twins are a cutting-edge approach to enhance vehicle security by creating virtual replicas of automotive systems and simulation environments [71]. In this context, security and privacy issues can be analyzed using a digital twin rather than a real vehicle [72]. This can help finding vulnerabilities and testing communication protocols [73], or even charging protocols for electric vehicles [74].
- Artificial Intelligence and Machine Learning: AI can improve threat and anomaly detection, and intrusion prevention in connected and autonomous vehicles [75]. Machine learning models can analyze vast amounts of real-time data to identify potential attacks before they cause any damage. Although training these models requires large, diverse datasets, AI can also generate synthetic data, which enables researchers to create simulation environments for a wide range of cyberattacks and rare threat scenarios [76]. Simulations allow for the continuous testing and validation of security measures in a controlled environment. This approach accelerates the development of automotive cybersecurity and ensures safety in future intelligent transportation systems.

## 5. Conclusions

In summary, this paper has presented a comprehensive analysis of the attack surface of modern vehicles, with a specific focus on wireless communication technologies. By examining the security risks associated with different wireless technologies, this study highlights potential vulnerabilities that attackers can exploit. Each area of study has been carefully analyzed, and relevant research articles and conference papers have been referenced to provide a strong foundation for further investigation into automotive cybersecurity.

As automotive manufacturers continue to integrate advanced technologies to improve safety, security, driving experience, and comfort, the complexity of automotive systems grows exponentially. This introduces new attack vectors that cybercriminals can leverage. It is a fact that vehicles still lack cybersecurity best practices. For instance, proprietary cryptographic protocols often exhibit weaknesses, infotainment systems are frequently exploited due to software vulnerabilities, and keyless entry systems remain susceptible to *script-kiddie* attacks such as replay and relay attacks, making them easy targets for car thieves. In addition, the growing adoption of V2X further expands the attack surface.

To mitigate these threats, automotive manufacturers must adopt the security-by-design principle, ensuring that cybersecurity is an integral part of vehicle development. This involves implementing strong cryptographic standards, continuous software updates, and intrusion detection systems capable of identifying and mitigating real-time threats. In addition, collaboration between vehicle manufacturers, cybersecurity researchers, and regulatory bodies is essential to establish cybersecurity best practices, as well as security audits, penetration testing, and coordinated vulnerability-disclosure programs. The introduction of stringent cybersecurity regulations, such as UN Regulations No. 155 and 156 [1,2], marks a significant step forward in ensuring that vehicles meet minimum security requirements. However, it is still not sufficient; continuous monitoring, risk assessment, and adaptation to emerging threats are necessary to maintain a secure automotive ecosystem.

## Data Availability

Data are contained within the article.
